# LncRNA LINC00667 aggravates the progression of hepatocellular carcinoma by regulating androgen receptor expression as a miRNA-130a-3p sponge

**DOI:** 10.1038/s41420-021-00787-4

**Published:** 2021-12-14

**Authors:** Zhixiang Qin, Xiaohong Liu, Zijing Li, Ganggang Wang, Zhe Feng, Ye Liu, Hai Yang, Chengpeng Tan, Zidong Zhang, Kun Li

**Affiliations:** 1 Department of Hepatobiliary and Pancreatic Surgery, Zhongnan Hospital of Wuhan University, Wuhan, 430071 China; 2 Department of Health Management and Policy, College for Public Health and Social Justice, St. Louis, MO USA; 3 Department of Health and Clinical Outcomes Research, Advanced Health Data Institute, School of Medicine, Saint Louis University, Saint Louis, MO USA

**Keywords:** Oncogenes, Cancer therapy, Hepatocellular carcinoma, Tumour biomarkers

## Abstract

Emerging studies have found long noncoding RNAs, widely expressed in eukaryotes, crucial regulators in the progression of human cancers, including hepatocellular carcinoma (HCC). Although the long intergenic noncoding RNA 667 (LINC00667) can promote the progression of a variety of cancer types, the expression pattern, the role in cancer progression, and the molecular mechanism involved in HCC remain unclear. This study aims to investigate the function and mechanism of LINC00667 in HCC progression. The effects of LINC00667 silencing in cell proliferation, cell migration, and cell invasion, and androgen receptor (AR) expression were determined with loss-of-function phenotypic analysis in Huh-7 and HCCLM3 cells, and subsequently testified in vivo in tumor growth. We found that the expression of LINC00667 was upregulated in HCC tissues and cell lines. Upregulation of LINC00667 was significantly associated with the unfavorable prognosis of HCC in our study patients. On the other hand, low expression of LINC00667 significantly inhibited the cell proliferation, cell migration and cell invasion of HCC in vitro and tumor growth in vivo. This inhibitory effect could be counteracted by miR-130a-3p inhibitor. LINC00667 reduced the inhibition of AR expression by miR-130a-3p, which correlated with the progression of HCC. Our finding suggests LINC00667 is a molecular sponge in the miR-130s-3p/AR signal pathway in the progression of HCC, in which it relieves the repressive function of miR-130a-3p on the AR expression. This indicates LINC00667 functions as a tumor promotor in promoting HCC progression through targeting miR-130a-3p/AR axis, making a novel biomarker and potential therapeutic target for HCC.

## Introduction

Hepatocellular carcinoma (HCC) is a common malignancy of the digestive system, especially in the developing world. Despite continuous advancement in diagnosis and treatment, the overall survivorship of HCC remains unsatisfactory, in which the five-year survival rate of early-stage HCC patients who have undergone surgery is only 61% [1]. Therefore, further exploration of the molecular mechanism of HCC and the development of more effective treatment strategies are of utmost urgency.

Long noncoding RNA (lncRNA) is a class of nonprotein-coding RNAs (>200 nt) that have multiple functions such as participating in protein translation, interacting with RNA-binding protein (RBP) and acting as a miRNA sponge [2–5]. Moreover, lncRNAs can regulate the occurrence and progression of various cancers, and have a variety of effects on biological processes, including cell proliferation (CP), cell metastasis (CM) and cell invasion (CI) [6]. A number of lncRNAs have been proven to be functioned in some ways in HCC cells like tumorigenesis [7], chemotherapy resistance [8] and treatment outcome [9]. Long intergenic noncoding RNA 667 (LINC00667) is a cancer-related lncRNA, and its abnormal expression levels have been widely reported in various kinds of cancers including cholangiocarcinoma [10], nephroblastoma [11], and esophageal cancer [12]. However, the exact molecular mechanisms of LINC00667 in HCC remain largely unknown.

MicroRNA (miRNA) is a class of ubiquitous and conservative small noncoding RNAs that modulate cellular physiology [13]. Numerous miRNAs actively participate in the occurrence and progression of HCC. For instance, miR-182-5p is associated with the progression of HCC by repressing FOXO3a [14]. However, some miRNAs can be targeted by lncRNAs through competitively interaction with miRNA response elements, thus inhibiting their function and expression [15]. Among these miRNAs, miRNA-130a-3p is found to be lowly expressed in HCC tissues and cells, which can serve as a potential therapeutic biomarker for HCC [16]. Therefore, it is of great interest to elucidate the regulatory roles of miRNA-130a-3p in HCC for developing new therapeutic strategies.

Androgen receptor (AR) is a ligand-dependent transcription factor that can control the expression of target genes upon binding to AR elements [17, 18]. Previous studies have reported that AR plays crucial roles in the progression of malignant neoplasms such as prostate, bladder, lung and breast cancers [19–22]. As for HCC, higher expression of AR was observed in HCC tissue than in adjacent liver tissue [23]. A previous study has revealed that AR can target the AR elements in the Slug promoter region, which in turn inhibits cell adhesion and promotes CM and CI in HCC [24]. Moreover, AR can upregulate the expression of EZH2 and CCRK, and subsequently activate β-catenin, thereby promoting tumor CP [25, 26]. Paradoxically, other studies have suggested that AR may promote the early development of HCC, but inhibit the metastasis of advanced HCC [27, 28]. Therefore, it is necessary to clarify the exact role and molecular mechanisms of AR in HCC.

Here, we determined the effect of LINC00667 on tumor progression in HCC tissues, and explored the molecular and functional mechanisms of LINC00667 pertaining to HCC progression. We hypothesized that LINC00667 could be upregulated in HCC tissues and acted as a miRNA-130a-3p sponge. In addition, the expression of AR, as well as the CP, CM, and CI of HCC cells should demonstrate the hypothesized effects of LINC00667 accordingly. It is hoped that our findings will imply the significance of LINC00667 as a potential therapeutic biomarker and provide novel insights into the prognostic prediction and treatment of HCC.

## Results

### LINC00667 is upregulated in HCC tissues and associated with poor prognosis

**Fig. 1:**
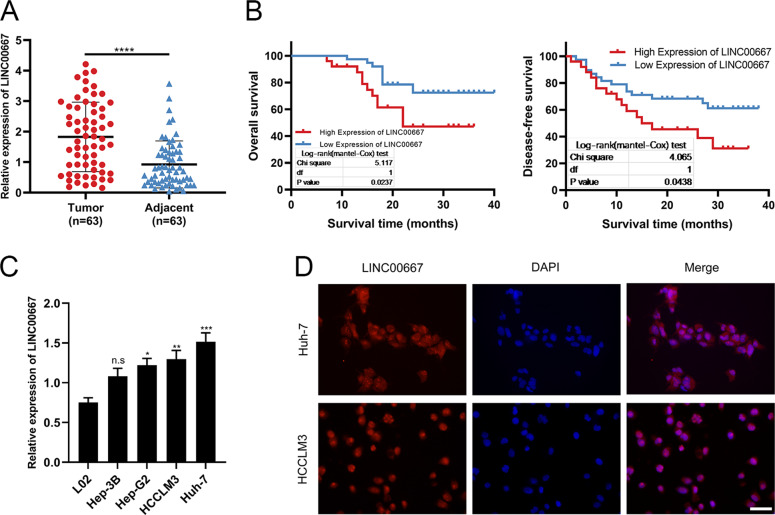
LINC00667 is overexpressed in HCC tissues and contributes to poor prognosis. **A** Overexpression of LINC00667 was detected in HCC tissues via RT-qPCR assay (*n* = 63, paired *t* test). **B** Kaplan-Meier DFS and OS analysis of HCC patients with downregulated and upregulated expression of LINC00667. **C** The relative expression of LINC00667 in HCC and normal L02 cell lines were detected by RT-qPCR. Data (*n* = 3) were analyzed by student’s *t* test. **D** Localization of LINC00667 in the cytoplasm and nucleus was detected by FISH assay. Blue color denotes DAPI-stained nuclei; red color denotes LINC00667; scale bar, 50 μm; magnification, 400×. Data, mean ± S.D.; ns, no significance. *, **, *** and **** = *P* < 0.05, *<*0.01, *<*0.001 and *<*0.0001, respectively.

To examine the roles of LINC00667 in HCC, we first detected the mRNA expression of LINC00667 in 63 HCC tissues relative to adjacent tissues (Fig. 1A). The expression of LINC00667 in HCC tissue was upregulated relative to adjacent tissue. Next, the HCC patients were categorized into two subgroups depending on the median expression level of LINC00667 as the cut-off value. We found that LINC00667 expression was highly correlated with several clinicopathological characteristics of 63 HCC tissues, namely, tumor size, TNM-stage, T grade and lymph node invasion (Table 1). Kaplan-Meier survival curves illustrated that the disease-free survival (DFS) and OS of HCC patients with upregulated LINC00667 expression were shorter (Fig. 1B). Besides, the expression levels of LINC00667 in Hep-3B, Hep-G2, HCCLM3, and Huh-7 cell lines relative to L02 cell line were confirmed (Fig. 1C). The localization of LINC00667 was observed in the cytoplasm and nucleus (Fig. 1D), indicating that LINC00667 may play a role in both cytoplasm and nucleus.

**Table 1:** Correlation between expression of LINC00667 and miR-130a-3p and clinicopathological characteristics in HCC tissues.

characteristics		LINC00667 expression		*p* value		miR-130a-3p expression		*p* value
	case	high	low			high	low	
all cases	63	25	38			18	45	
age groups				0.793				0.062
≥60	29	11	18			5	24	
<60	34	14	20			13	21	
gender				0.328				0.621
male	49	21	28			14	35	
female	14	4	10			4	10	
TNM-stage				**0.009**				0.077
I	22	3	19			7	15	
II	14	6	8			6	8	
III	11	6	5			4	7	
IV	16	10	6			1	15	
T grade				**0.002**				0.377
T1 + T2	44	12	32			14	30	
T3 + T4	19	13	6			4	15	
lymph node invasion				**0.032**				**0.011**
N0	47	15	32			17	30	
N1	16	10	6			1	15	
histological grade				0.198				0.47
low	34	11	23			11	23	
middle-high	29	14	15			7	22	
tumor size				**0.005**				**<0.001**
≥5	40	21	19			4	36	
<5	23	4	19			14	9	

*TNM* tumor-node-metastasis.

### LINC00667 promotes CP, CM, and CI in HCC cells

**Fig. 2:**
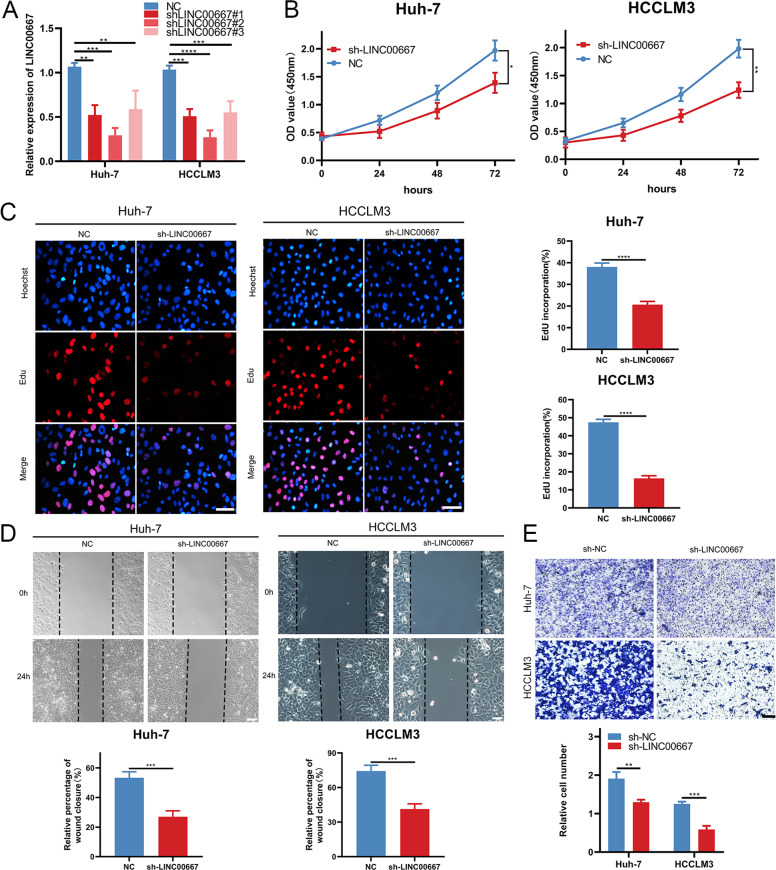
LINC00667 aggravates CP, CM and CI in HCC cells. **A** Relative expression of LINC00667 was silenced by 3 shRNAs in HCCLM3/Huh-7 cells. Data (*n* = 3) were analyzed by student’s *t* test. **B** and **C** Proliferative abilities of sh-LINC00667 or sh-NC-transfected HCCLM3/Huh-7 cells were evaluated by CCK-8 and EdU assays, respectively. Scale bar, 100 μm. The result was analyzed by student’s *t* test. **D** Migratory capabilities of sh-LINC00667 or sh-NC-transfected HCCLM3/Huh-7 cells were determined by wound healing assays. Scale bar, 100 μm. Data were analyzed by student’s *t* test. **E** Invasive capabilities of sh-LINC00667 or sh-NC-transfected HCCLM3/Huh-7 cells were determined by transwell assays. Scale bar, 150 μm. Data were analyzed by student’s *t* test. Data, mean ± S.D. *, **, *** and **** = *P* < 0.05, *<*0.01, *<*0.001 and *<*0.0001, respectively.

To investigate the functionality of LINC00667 in HCC cells, we constructed 3 shRNAs targeting LINC00667 (shLINC00667#1-3) with nontargeting shRNA as negative control (NC) in HCCLM3 and Huh-7 cells to silence LINC00667. Notably, shLINC00667#2 was selected for follow-up experiments because shLINC00667#2 exhibited the highest suppression efficiency (Fig. 2A). CCK-8 assay showed that the downregulation of LINC00667 significantly attenuated the proliferative ability of HCC cells (Fig. 2B). EdU assays demonstrated that silencing LINC00667 markedly decreased the proportion of EdU-positive cells (Fig. 2C), suggesting that LINC00667 could promote CP in HCC cells. In addition, the effects of LINC00667 on CM and CI in HCC cells were evaluated. The results showed that LINC00667 knockdown significantly weaken the migration and invasion capabilities of HCCLM3/Huh-7 cells (Fig. 2D, E), which implied that LINC00667 could promote CP, CM, and CI in HCC cells.

### miRNA-130a-3p is downregulated in HCC tissues and suppresses CP, CM and CI in HCC cells

**Fig. 3:**
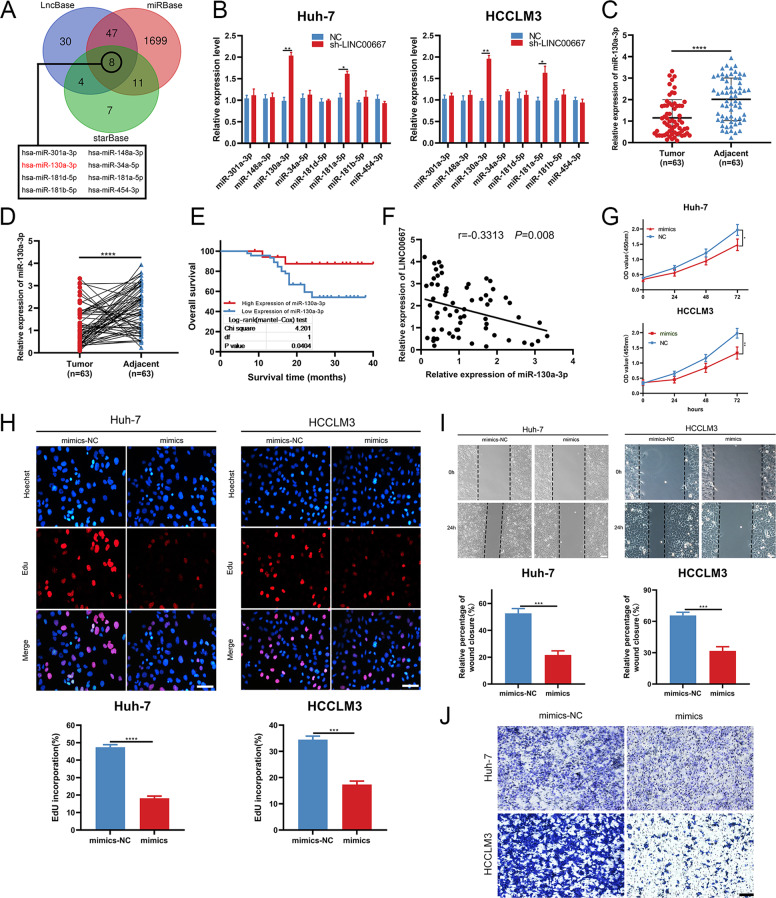
miRNA-130a-3p suppresses CP, CM and CI in HCC cells. **A** Using starBase, LncBase and miRBase, LINC00667 was targeted by 8 miRNAs. **B** Relative expression of candidate miRNAs in HCCLM3 and Huh-7 cells, miRNA-130a-3p was selected for further analyses. Data (*n* = 3) were analyzed by student’s *t* test. **C** and **D** Expression levels of miRNA-130a-3p in HCC and paired adjacent tissues were determined by RT-qPCR assay (*n* = 63, paired *t* test). **E** Kaplan-Meier OS analysis of HCC patients with downregulated and upregulated miRNA-130a-3p expression. **F** Pearson’s correlation coefficients of the relationship between LINC00667 and miRNA-130a-3p expression in HCC tissues. **G** and **H** Proliferative abilities of HCCLM3/Huh-7 cells transfected with mimics or mimics-NC were evaluated by CCK-8 and Edu assays, respectively. Scale bar, 100 μm. Data were analyzed by student’s *t* test. **I** Migratory capabilities of HCCLM3/Huh-7 cells transfected with mimics or mimic-NC were determined by wound healing assays. Scale bar, 100 μm. Data were analyzed by student’s *t* test. **J** Invasive capabilities of HCCLM3/Huh-7 cells transfected with mimics or mimic-NC were determined by transwell assays. Scale bar, 150 μm. Data, mean ± S.D. *, **, *** and **** = *P* < 0.05, *<*0.01, *<*0.001 and *<*0.0001, respectively.

Considering that LINC00667 is localized in the cytoplasm and based on the concept of competing endogenous RNA (ceRNA), we further investigated whether LINC00667 can promote the malignant behavior of HCC tissues by acting as miRNA sponges [29]. The putative targets of LINC00667 were predicted by computational target prediction tools such as LncBase, starBase and miRBase [30–32]. After overlapping the obtained results from the three prediction tools, eight candidate miRNAs were identified and selected for further verification (Fig. 3A). We detected the relative expression levels of candidate miRNAs in HCCLM3/Huh-7 cells transfected with sh-LINC00667. The findings demonstrated that the expression levels of miRNA-130a-3p and miRNA-181a-5p were remarkably elevated compared to other candidate miRNAs (Fig. 3B). Then, the expression levels of miRNA-130a-3p and miRNA-181a-5p in liver cancer tissues and normal tissues were obtained through the TCGA database. The findings revealed that the expression of miRNA-130a-3p was markedly lower in liver tumor tissue than in normal tissue, while that of miRNA-181a-5p was higher in liver tumor tissue (Fig. S1A, B).

The role of miRNA-130a-3p in HCC progression was further clarified. From the RT-qPCR results in the aforementioned 63 pairs of tissues, we observed that the expression of miRNA-130a-3p in HCC tissue was dramatically reduced compared to the paired adjacent tissues, and the expression of miRNA-130a-3p was positively associated with HCC prognosis (Fig. 3C–E), which demonstrated a negative correlation between LINC00667 and miRNA-130a-3p (Fig. 3F). In addition, the expression of miRNA-130a-3p was significantly related to lymph node invasion and tumor size (Table 1). Therefore, miRNA-130a-3p can serve as a tumor inhibitory factor of HCC, and LINC00667 probably regulates the biological behavior of HCC through sponging miRNA-130a-3p.

We further assessed the function of miRNA-130a-3p in HCC cells. CCK-8 assay revealed that overexpression of miRNA-130a-3p remarkably reduced the proliferative activity of HCC cells (Fig. 3G). EdU assay demonstrated that the upregulation of miRNA-130a-3p significantly reduced the proportion of EdU-positive cells (Fig. 3H). These data showed that miRNA-130a-3p suppressed CP in HCC cells. In addition, the effects of miRNA-130a-3p on CM and CI in HCC cells were determined. The findings revealed that increased expression of miRNA-130a-3p tremendously inhibited the migration and invasion capabilities of HCCLM3/Huh-7 cells (Fig. 3I, J). This suggests that miRNA-130a-3p can inhibit CP, CM, and CI in HCC cells.

### AR is a novel target of miRNA-130a-3p

**Fig. 4:**
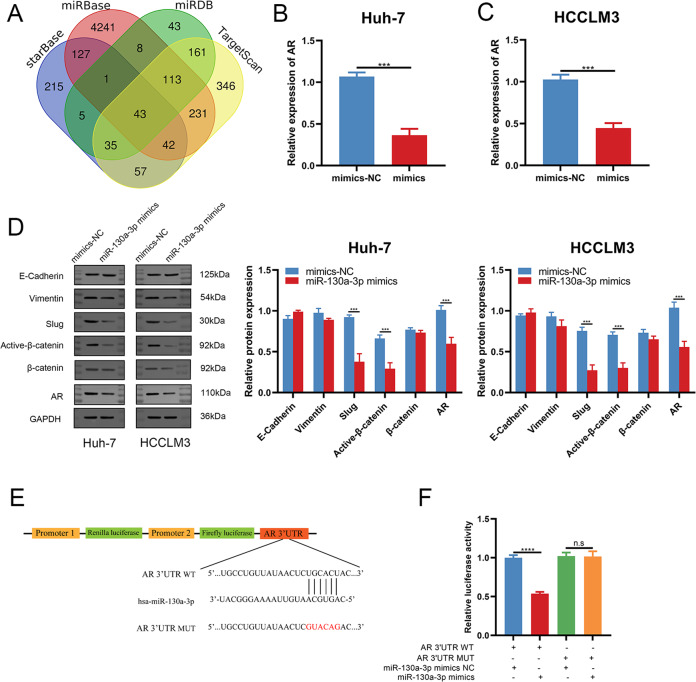
AR is directly targeted by miRNA-130a-3p. **A** Using starBase, miRBase, miRDB and TargetScan, we predicted 43 target genes of miRNA-130a-3p. **B** and **C** Relative expression of AR in HCCLM3/Huh-7 cells transfected with miRNA-130a-3p mimic was evaluated by RT-qPCR assay. Data (*n* = 3) were analyzed by student’s *t* test. **D** Protein levels of AR, Slug and active-β-catenin in cells transfected with miRNA-130a-3p mimic and NC were detected by immunoblotting. **E** and **F** Complementary sequences of miRNA-130a-3p and AR-WT. The putative binding sites of miRNA-130a-3p was mutated in AR-MUT. 293 T cells co-transfected with miRNA-130a-3p mimic and AR-WT or AR-MUT vector were respectively detected for luciferase activity. Data (*n* = 3) were analyzed by student’s t test. Data, mean ± S.D.; ns, no significance. *** and **** = *P* < 0.001 and *<*0.0001, respectively.

To further clarify the mechanistic action of miRNA-130a-3p in HCC cells, we explored the target genes of miRNA-130a-3p based on the prediction results of TargetScan, starBase, miRBase and miRDB (Fig. 4A) [31–34]. All candidate target genes were presented in Supplementary Table 1. Among the candidate target genes, only AR had been more frequently reported to be associated with the progression of HCC. Therefore, we chose AR as the target gene of miRNA-130a-3p for further experiment. Then, an HCC transcriptome dataset obtained from the Oncomine database (https://www.oncomine.org/) was used to analyze the expression of AR [35, 36]. This dataset demonstrated that AR expression was higher in HCC samples (Fig. S2). We respectively transfected miRNA-130a-3p mimic into Huh-7/HCCLM3 cells. The mRNA expression of AR was markedly reduced in miRNA-130a-3p mimic group in both Huh-7/HCCLM3 cell lines (Fig. 4B, C). Immunoblot results showed that AR expression in miRNA-130a-3p mimic group was downregulated, while Slug and active-β-catenin expression were also downregulated (Fig. 4D). AR-WT or AR-MUT cloned into the luciferase reporter vector pmirGLO was separately co-transfected with miRNA-130a-3p mimic in 293 T cells, and the interrelationship between miRNA-130a-3p and AR was analyzed (Fig. 4E). The results demonstrated that the luciferase activities of AR 3’UTR-WT were noticeably decreased in miRNA-130a-3p mimic group, but these phenomena were not found in AR 3’UTR-MUT group (Fig. 4F).

### LINC00667 promotes CP, CM, and CI in HCC cells through LINC00667/miRNA-130a-3p/AR axis

**Fig. 5:**
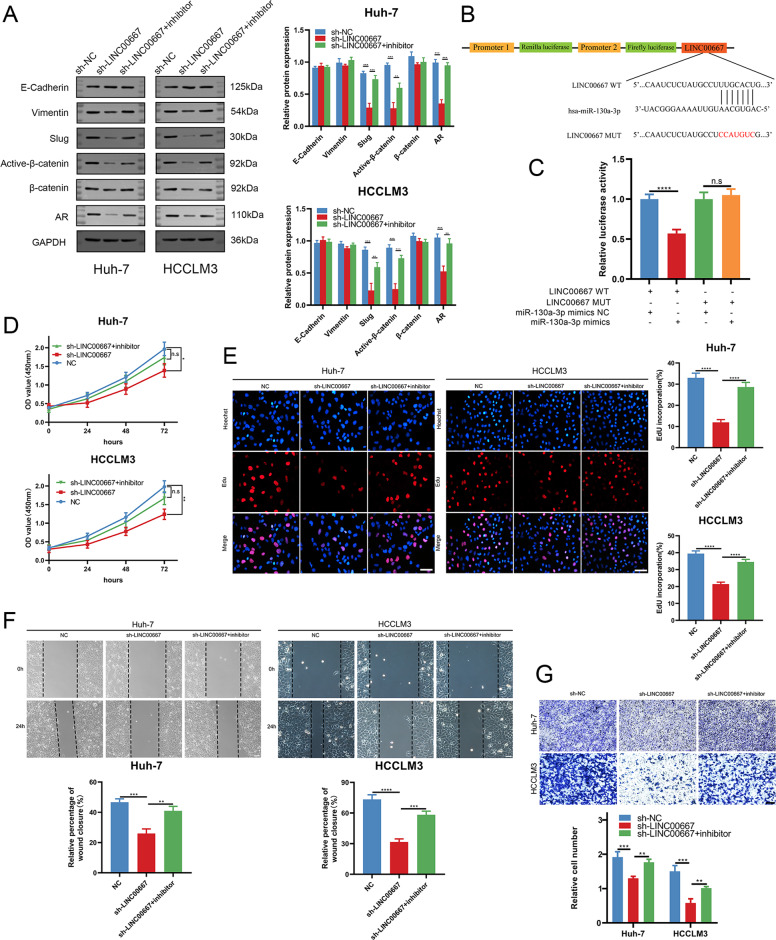
LINC00667 aggravates CP, CM and CI in HCC cells via LINC00667/miRNA-130a-3p/AR axis. **A** Relative protein levels of AR, Slug and active-β-catenin in HCCLM3/Huh-7 cells transfected with sh-LINC00667, sh-NC or sh-LINC00667 + miRNA-130a-3p inhibitor were evaluated by immunoblotting. **B** and **C** Complementary sequences of miRNA-130a-3p and LINC00667-WT. The putative binding sites of miRNA-130a-3p was mutated in LINC00667-MUT. 293 T cells co-transfected with miRNA-130a-3p mimic and LINC00667-WT or LINC00667-MUT vector were respectively detected for luciferase activity. Data (*n* = 3) were analyzed by student’s t test. **D** and **E** Proliferative abilities of HCCLM3/Huh-7 cells transfected with sh-LINC00667, NC or sh-LINC00667 + miRNA-130a-3p inhibitor were determined by CCK-8 and EdU assays, respectively. Scale bar, 100 μm. Data were analyzed by one-way ANOVA. **F** Migratory capabilities of HCCLM3/Huh-7 cells transfected with sh-LINC00667, NC or sh-LINC00667 + miRNA-130a-3p inhibitor were determined by wound healing assays. Scale bar, 100 μm. Data were analyzed by one-way ANOVA. **G** Invasive capabilities of HCCLM3/Huh-7 cells transfected with sh-LINC00667, NC or sh-LINC00667 + miRNA-130a-3p inhibitor were determined by transwell assays. Scale bar, 150 μm. Data were analyzed by one-way ANOVA. Data, mean ± S.D.; ns, no significance. *, **, *** and **** = *P* < 0.05, *<*0.01, *<*0.001 and *<*0.0001, respectively.

We designed rescue experiments using miRNA-130a-3p inhibitor to verify whether LINC00667 can promote tumor via LINC00667/miRNA-130a-3p/AR axis. Immunoblot results demonstrated that silencing LINC00667 reduced the protein levels of AR in Huh-7 cells and HCCLM3 cells, while those of Slug and active-β-catenin were also reduced. In addition, the effects of silencing LINC00667 were reversed by miRNA-130a-3p inhibitor (Fig. 5A). Then, LINC00667-WT and LINC00667-MUT were cloned into the pmirGLO and co-transfected with miRNA-130a-3p mimic in 293 T cells to confirm the interrelationship between miRNA-130a-3p and LINC00667 (Fig. 5B). The findings demonstrated that the luciferase activities of LINC00667-WT were remarkably decreased in miRNA-130a-3p mimic group, but these phenomena were not found in LINC00667-MUT group (Fig. 5C).

We further examined whether miRNA-130a-3p inhibitor can reverse the functional effect of LINC00667 on HCC cells. It was found that miRNA-130a-3p inhibitor reversed the suppression effects of CP, CM and CI in HCC cells caused by LINC00667 knockdown through CCK-8, EdU, wound healing and transwell assays (Fig. 5D–G). Altogether, these results demonstrate that LINC00667 regulates the expression of AR by acting as a miRNA-130a-3p sponge to fuel HCC progression.

### LINC00667 knockdown suppresses the growth of HCC in vivo

**Fig. 6:**
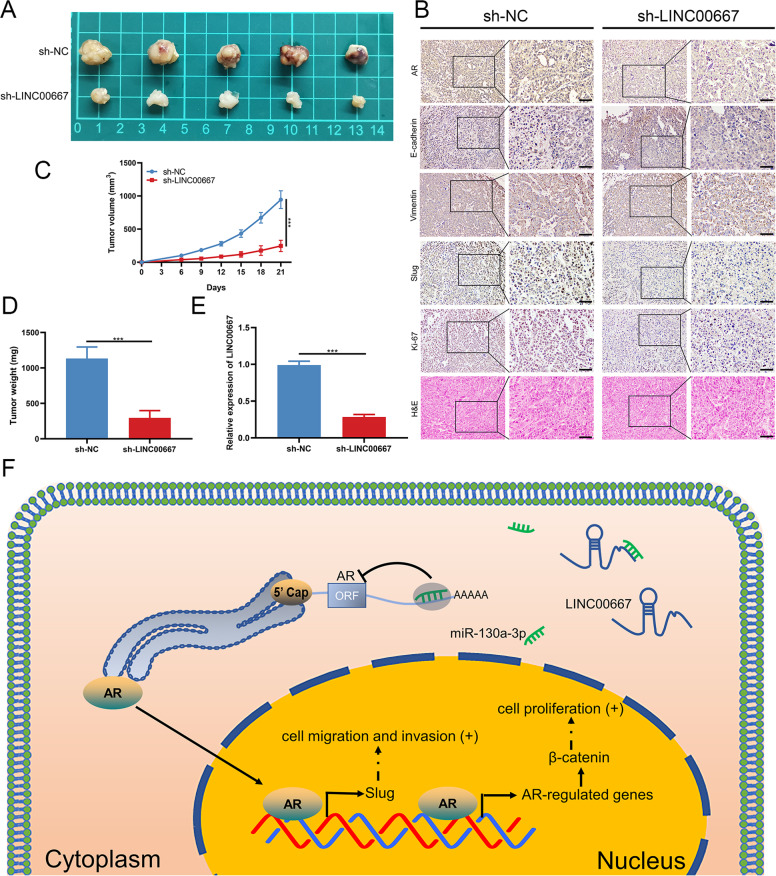
LINC00667 knockdown suppresses the growth of HCC in vivo. **A** Nude mice were injected subcutaneously with sh-LINC00667 or sh-NC-transfected HCCLM3 cells (*n* = 5 per group, student’s t test). Image of STTs in sh-LINC00667 and sh-NC groups. **B** Relative expression levels of AR, E-cadherin, Vimentin, Slug and Ki-67 in STTs were detected by immunostaining. HE staining image of STTs in sh-LINC00667 and sh-NC groups. Scale bar, 100 μm. **C** Tumor volume curves of mice were monitored every 3 days. Data were analyzed by student’s t test. **D** The subcutaneous tumor weights were determined at the 21st day. Data were analyzed by student’s t test. **E** Relative expression of LINC00667 in STTs was detected by RT-qPCR. Data were analyzed by student’s t test. **F** Schematic diagram showing the mechanisms by which LINC00667 acts as a miRNA-130a-3p sponge to promote AR expression during HCC progression. Data, mean ± S.D. ***P < 0.001.

To elucidate the in vivo effect of LINC00667 on HCC growth, HCCLM3 cells with LINC00667 knockdown were subcutaneously injected into nude mice. Tumor volume was recorded every 3 days, and tumor weight was determined after 21 days. The tumor volume curve and tumor weight showed that LINC00667 knockdown significantly reduced the growth of HCC tumor in mice (Fig. 6A, C, D). The collected tumor tissues were subjected to RT-qPCR and IHC analyses. The RT-qPCR data revealed that the expression of LINC00667 was remarkably lower in tumor tissue obtained from the sh-LINC00667 group compared to the control group (Fig. 6E). IHC assays proved that the downregulation of LINC00667 weakened the expression levels of AR, Slug and Ki-67 in tumor tissues (Fig. 6B). These results not only demonstrated that LINC00667 knockdown inhibited HCC tumor growth in vivo, but also indicated that LINC00667 acted as a miRNA-130a-3p sponge to promote HCC progression by upregulating AR expression (Fig. 6F).

## Discussion

Long noncoding RNAs are involved in the biological process of various tumors, including HCC. For example, lncRNA AY promoted HCC metastasis by inducing ITGAV transcription [37], and lncRNA-PDPK2P promoted the progression of HCC via PDK1/AKT/caspase 3 axis [38]. It has been reported that LncRNA LINC00667 is upregulated in many kinds of cancers such as colorectal cancer [39] and lung cancer [40] as well as nasopharyngeal carcinoma [41]. However, the roles of LINC00667 in HCC remain largely unclear.

Here, we demonstrated LINC00667 could regulate the cellular proliferation and migration of HCC both in vitro and in vivo, and delineated the signaling pathway responsible for the interaction of LINC00667 with AR and miRNA-130a-3p. These molecular data are consistent with the findings of lymph node invasion, tumor size and OS of HCC patients, which imply that LINC00667, as a promoting factor, can enhance CP, CM, and CI in HCC cells. Moreover, we observed that miRNA-130a-3p, a highly sensitive miRNA sequence identified by bioinformatics tool, was remarkably downregulated in HCC tissue and markedly related to the OS of HCC patients as well as CP, CM and CI in HCC cells. These findings altogether indicated the oncogenic effect of LINC00667 and the signaling pathway involved in HCC progression.

To date, it has been suggested that lncRNAs can play a vital role in the cytoplasm as miRNA sponges and participate in protein translation [42]. For instance, overexpression of LINC00963 in prostate cancer triggered the NOP2-induced EMT pathway by reducing miRNA-542-3p expression, thereby promoting tumor progression [43]. Besides, it was found that lncRNA ADAMTS9-AS2 was significantly reduced in gastric cancer, thus inhibiting the development of gastric cancer via miRNA-223-3p/NLRP3 axis [44]. Thus, LINC00667 was predicted to contain the binding sites of miRNA-130a-3p through LncBase, miRBase and starBase. FISH and dual-luciferase reporter assay revealed that LINC00667 was localized in both cytoplasm and nucleus of HCC cells and could bind to miRNA-130a-3p directly. The rescue experiments demonstrated that miRNA-130a-3p inhibitor could reverse the tumor-suppressive effect of LINC00667 knockdown. Thus, we believe that LINC00667 may act as a miRNA-130a-3p sponge.

Previous reports have indicated that HCC can occur in men and women at any age group, but the incidence in men is approximately 7 times higher than that in women [45]. AR is a male hormone receptor and also known as an oncogene of various cancers, including HCC. It has been reported that AR is positively correlated with pathological tumor staging in HCC patients [23]. However, other studies have suggested that AR can inhibit advanced HCC [27, 28]. Therefore, the actual role of AR in the development of HCC remains controversial. Nevertheless, our results showed that AR exhibited a cancer-promoting effect on HCC. It is possible that the tumor microenvironment in different stages or different types of cells can lead to this phenomenon. Consequently, it is necessary to further investigate the role of AR in progression of tumors.

To our knowledge, this study is the first to assess the expression, regulatory mechanism, functions and clinical significance of LINC00667 in HCC. However, there are some limitations that need to be addressed. First, the HCC tissues were derived from a homogenous population in the same hospital, which might bring selection bias and undermine the generalizability of the results. Second, although miRNA-130a-3p exhibited the highest sensitivity among the candidate miRNAs, some other miRNAs may also interact with LINC00667 and possibly have better effects on the progression of HCC. Third, whether LINC00667 directly participates in mRNA post-transcriptional regulation during the development of HCC has yet to be investigated. Therefore, the therapeutic potential of LINC00667 in HCC needs to be further explored.

## Materials and methods

### Patient samples

Liver cancer and the paired adjacent tissues were sampled from 63 patients who had undergone hepatectomy at Zhongnan Hospital of Wuhan University between 2015 and 2017. All patients were diagnosed with HCC via histopathological examination. After surgical resection, the tissues were snap-frozen in liquid nitrogen and stored at −80 °C. The ethical approval for this clinical research was obtained from the ethics committee of Zhongnan Hospital of Wuhan University. All volunteers signed an informed consent before study enrollment.

### Cell maintenance

The human immortalized hepatocytes L02 and human HCC cells (Huh-7, HCCLM3, Hep-3B and Hep-G2) were all procured from the Cell Bank of Type Culture Collection (CBTCC, China). All cells were maintained in high-glucose DMEM (Gibco, USA) supplemented with fetal bovine serum (FBS, 10%; Gibco, USA) and antibacterial drugs (1% penicillin-streptomycin; Gibco, USA) at 37 °C and 5% CO_2_ under a humidified condition.

### Real-time quantitative polymerase chain reaction (RT-qPCR)

Trizol reagent (Invitrogen, USA) was utilized to isolate total RNA from HCC cell lines/tissues and paired adjacent tissues. For LncRNA and mRNA, reverse transcription was performed with HisScript III 1st Strand cDNA Synthesis Kit (Vazyme, China). For miRNA, cDNA was synthesized with PrimeScript RT Reagent Kit (Takara, Japan) containing specific stem-loop primers. RT-qPCR was conducted on a CFX Connect RT-PCR Detection System (Bio-Rad, USA) using SYBR RT-qPCR Mix (Toyobo, Japan). The expression activities of lncRNA, miRNA and mRNA were determined using the 2^−ΔΔCT^ approach and U6/GAPDH was employed as a reference gene. The primer sequence list is demonstrated in Supplementary Table 2.

### Cell transfection

The lentiviral small hairpin RNAs (shRNAs) expression vector targeting LINC00667 gene (shLINC00667#1-3) and negative control shRNA (sh-NC) were designed and produced by Genecreate (Wuhan, China). Each shRNA was stably transfected into HCCLM3 and Huh-7 cell lines. MiRNA-130a-3p inhibitor, miRNA-130a-3p mimic and negative control were all purchased from Genecreate (Wuhan, China). Lipofectamine 2000 reagent (Thermo-Fisher-Scientific, USA) was employed to transfect the plasmids into HCC cells by following the manufacturer’s protocol.

### Fluorescence in situ hybridization (FISH)

The localization of LINC00667 in HCC cells was examined with FISH assay. The Cy3-labeled LINC00667 probes were designed and produced by Servicebio (Wuhan, China). As per the manufacturer’s instructions, hybridization was performed overnight with LINC00667 probes at 42 °C. After the hybridization got washed off, each section was counterstained with DAPI (Servicebio, China). These images were recorded and collected under an upright fluorescence microscope (Nikon, Japan). The sequences of the LINC00667 probe are presented in Supplementary Table 2.

### Luciferase reporter assay

LINC00667 sequences and AR 3′UTR and their respective mutations were designed, generated and transferred into the pmirGLO vectors (Genecreate, China). Each plasmid was respectively co-transfected with miRNA-130a-3p mimic in 293 T cells. The luciferase activities were determined with Dual-Luciferase Reporter Gene Assay Kit (Beyotime, China) by following the manufacturer’s protocol, and then normalized by Renilla activities.

### CP assays

The viability of HCC cell lines in a 96-well plate was examined by CCK-8 assay. HCC cell suspensions (1 × 10^4^/ml) were plated in each well, and 10 μL of CCK-8 solution (Dojindo Crop, Japan) was added at each time point (0, 24, 48 or 72 h). After incubation at 37 °C for 2 h, the optical density of each sample was detected using a microplate reader (450 nm; Thermo-Fisher-Scientific, USA).

The CP of HCC cells was also determined by EdU assay using Cell-Light EdU Apollo567 In Vitro Kit (RiboBio, China). HCCLM3 and Huh-7 cells were cultured in 6-well plates for 24 h, followed by the addition of 50 uM EdU solution. After incubation at 37 °C for 2 h, the cell lines were fixed with paraformaldehyde (4%) and successively stained with Apollo Dye Solution and Hoechst 33342. Finally, the cells were examined using the IX51 inverted microscope (Olympus, Japan).

### Wound healing assay

The CM of HCC cells was assessed by wound healing assay. Briefly, HCC cells were grown in 6-well plates, and then scratched by a sterile 100-µl pipette tip. Typical images of CM were taken at 0 and 24 h to measure the migrated distance. The relative migration rate was obtained based on the reduction of the distance through the scratched area after normalization to the respective control value at 0 h.

### Transwell invasion assay

The CI of HCC cells was determined by Transwell chambers precoated with matrigel (BD Biosciences, USA). HCC cells cultured without serum placed into the top chamber, while the culture medium with 10% FBS was placed into the bottom chamber. After fixing in 4% paraformaldehyde, a crystal violet solution was utilized to stain the invaded cells.

### Immunoblot analysis

HCC cells were lysed with RIPA buffer (Beyotime, China) containing phosphatase/protease inhibitors (Roche, Germany), and total protein was detected with a BCA protein assay (Beyotime, China). Proteins were transferred onto polyvinylidene difluoride (PVDF) after being electrophoresed through 10% SDS-PAGE. After blocking with 5% skimmed milk, the protein samples were exposed to specific primary antibodies at 4 °C overnight. The membranes were rinsed 3 times and then incubated with HRP-conjugated secondary antibodies for 2 h. Lastly, the Clarity Western ECL Substrate (Bio-Rad, USA) was used to identify antibody-bound proteins. The information of each antibody used is presented in Supplementary Table 3.

### Xenografts in mice

BALB/c nude mice (male, 5-week-old) were supplied by the Central Laboratory of Animal Science (Wuhan University, China) and randomly divided them into same two groups for xenograft experiments. HCCLM3 cells stably transfected with sh-LINC00667or sh-NC were injected subcutaneously into the armpit of nude mice (*n* = 5 per group). Tumor volume was recorded every 3 days using a vernier caliper, and then measured as (length × width^2^)π/6. Following three weeks, the mice were sacrificed under anesthesia, and their subcutaneous tumor tissues (STTs) were used for the measurement of tumor weight.

### Immunochemistry (IHC) examination

After fixing in paraformaldehyde (4%), embedding in paraffin and sectioning at 4-μm thick, the tissue samples were exposed to anti-AR, anti-E-cadherin, anti-Vimentin, anti-Ki-67 or anti-Slug primary antibody at 4 °C overnight. Subsequently, the tissue samples were exposed to the corresponding HRP-conjugated secondary antibodies. The information of each antibody used is presented in Supplementary Table 3.

### Hematoxylin-eosin (HE) staining

The tumor samples were collected from mice, followed by fixing in paraformaldehyde (4%), embedding in paraffin and sectioning. After HE staining, the samples were examined using a light microscope (Olympus, Japan).

### Statistical analysis

All results were presented as mean ± standard deviation (MEAN ± SD). SPSS v22.0 (IBM, USA) and GraphPad Prism v8.0 were employed to perform the statistical tests. Significant differences between groups were compared by student’s *t* test or one-way ANOVA. Pearson correlation coefficient was used to measure the correlation between two variables. Kaplan-Meier method was employed to assess the overall survival (OS) rates, and the difference was compared using the log-rank test. A *p* value of <0.05 was deemed statistically significant.

## Supplementary information

Supplemental Material

Supplementary figure legends

Supplementary Figure 1

Supplementary Figure 2

Supplementary Table 1

Supplementary Table 2

Supplementary Table 3

## Data Availability

The datasets used or analyzed during the study are available from the corresponding author on reasonable request.
