# Supplementary material for: LncRNA LINC00667 aggravates the progression of hepatocellular carcinoma by regulating androgen receptor expression as a miRNA-130a-3p sponge
Source: Cell Death Discov. 2021 Dec 14;7:387. doi: 10.1038/s41420-021-00787-4 (PMC8671440; doi:10.1038/s41420-021-00787-4)
Supplement: Supplementary file 1 — Supplemental Material [file 41420_2021_787_MOESM1_ESM.docx]

**Supplementary Figure 1**


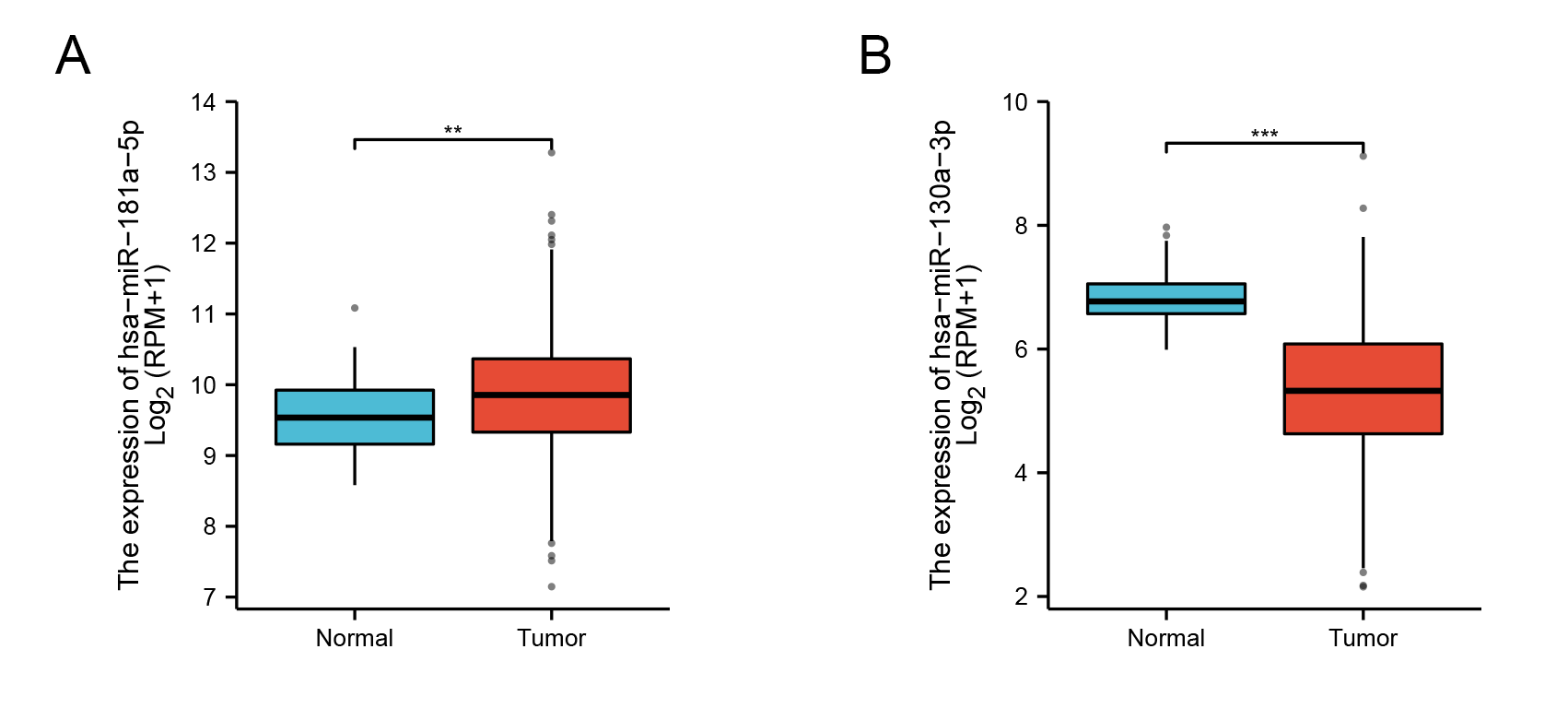


**Fig. S1** The expression levels of miRNA-130a-3p and miRNA-181a-5p in liver cancer tissues and normal tissues. **a** The expression of miRNA-181a-5p in liver cancer tissues and normal tissues was obtained through TCGA database. **b** The expression of miRNA-130a-3p in liver cancer tissues and normal tissues was obtained through TCGA database. ** and *** = *P* *<*0.01 and *<*0.001.

**Supplementary Figure 2**


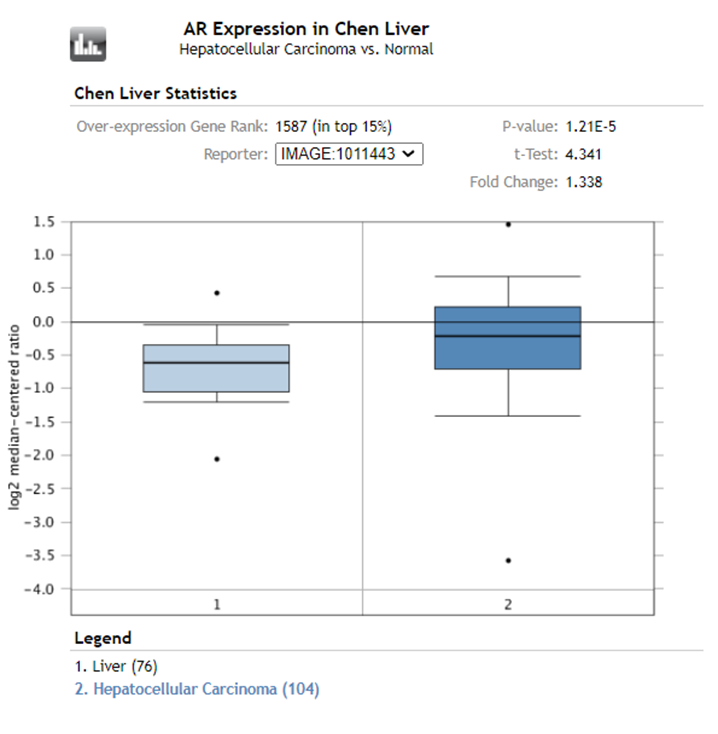


**Fig. S2** The expression of AR in HCC tissues and normal tissues was obtained through Oncomine database. *P* <0.001.

**Supplementary Table 1. Candidate target genes of miRNA-130a-3p**

| SLMAP | RPS6KA5 | ACBD3 | SEL1L3 | UBE2W | CUL3 | ACSL1 | CLCN3 | SYBU |
| --- | --- | --- | --- | --- | --- | --- | --- | --- |
| MFSD6 | ACBD5 | NACC2 | PRKAA1 | SNX2 | PRUNE2 | PLAA | PSAP | AKAP1 |
| ZNF800 | RRAGD | AR | TRIM2 | CDS1 | ZBTB18 | HSPA8 | ITPRIPL2 | DNAJC16 |
| TEX2 | ARHGAP12 | ZBTB4 | MAPK1 | G3BP2 | UBE3B | SPTY2D1 | IMPDH1 | AKAP11 |
| INO80 | BHLHE41 | PPARG | SLC44A1 | UBXN2B | VPS13D | CLTC |  |  |

**Supplementary Table 2. Primer sequences and probe sequences**

| **Name** | **Sequence** |
| --- | --- |
| LINC00667-F | 5′-GTGGGTAGGAAACAGTCGGG-3′ |
| LINC00667-R | 5′-CTCAAAGGTGGCCAAAAGCC-3′ |
| GAPDH-F | 5′-GTCTCCTCTGACTTCAACAGCG-3′ |
| GAPDH-R | 5′-ACCACCCTGTTGCTGTAGCCAA-3′ |
| miR-130a-3p-F | 5'-TTCACATTGTGCTACTGTCTGC-3' |
| miR-130a-3p-R | 5'-GCTCTGACTTTATTGCACTACT-3' |
| U6-F | 5'-CTCGCTTCGGCAGCACA-3' |
| U6-R | 5'-AACGCTTCACGAATTTGCGT-3' |
| miR-130a-3p mimics | 5'-CAGUGCAAUGUUAAAAGGGCAU-3' |
| miR-130a-3p inhibitor | 5'-AUGCCCUUUUAACAUUGCACUG-3' |
| mimics-NC | 5'-UUCUCCGAACGUGUCACGUTT-3' |
| AR-F | 5'-TTCCCATTGTGGCTCCTATC-3' |
| AR-R | 5'-GTGGCTGGCACAGAGTAGTG-3' |
| miR-301a-3p-F | 5'-ACACTCCAGCTGGGCAGTGCAATAGTATTGTC-3' |
| miR-301a-3p-R | 5'-CTCAACTGGTGTCGTGGA-3' |
| miR-148a-3p-F | 5′-AGCAGTTCAGTGCACTACAG-3′ |
| miR-148a-3p-R | 5′-GCAGGGTCCGAGGTATTC-3′ |
| miR-181d-5p-F | 5′-GCAAACATTCATTGTTGTCGGT-3′ |
| miR-181d-5p-R | 5'-CCAGTGCAGGGTCCGAGGT-3' |
| miR-34a-5p-F | 5′-AGCCGCTGGCAGTGTCTTA-3′ |
| miR-34a-5p-R | 5'-CAGAGCAGGGTCCGAGGTA-3' |
| miR-181a-5p-F | 5'-GGGCAGCCTTAAGAGGA-3' |
| miR-181a-5p-R | 5'-CAGTGCGTGTCGTGGA-3' |
| miR-181b-5p-F | 5'-AACATTCATTGCTGTCGGTGGGT-3' |
| miR-181b-5p-R | 5'-GCGAGCACAGAATTAATACGAC-3' |
| miR-454-3p-F | 5'-GCGCGTAGTGCAATATTGCTTA-3' |
| miR-454-3p-R | 5'-AGTGCAGGGTCCGAGGTATT-3' |
| LINC00667 probe | 5'CY3-CAGGACGGGGCTCTCCCTCTCGATGT-3' |
|  |  |

**Supplementary Table 3. Antibodies used in this study**

| **Antibody** | **WB** | **IHC** | **Specificity** | **Company** |
| --- | --- | --- | --- | --- |
| AR (22089-1-AP) | 1:1000 | 1:100 | Mouse polyclonal | Proteintech |
| GAPDH (ab8245) | 1:5000 |  | Mouse monoclonal | Abcam |
| Slug (GTX128796) | 1:5000 | 1:200 | Rabbit polyclonal | GeneTex |
| Active-β-Catenin (05-665) | 1:1000 | 1:100 | Mouse monoclonal | MERCK |
| β-Catenin (ab32572) | 1:5000 | 1:500 | Mouse monoclonal | Abcam |
| E-cadherin (bs-1519R) | 1:500 | 1:200 | Rabbit polyclonal | Bioss |
| Vimentin (ab92547) | 1:2000 | 1:400 | Rabbit monoclonal | Abcam |
| Ki67 (NBP2-22112) |  | 1:400 | Mouse monoclonal | Novus |
