# Supplementary material for: LncRNA LINC00667 aggravates the progression of hepatocellular carcinoma by regulating androgen receptor expression as a miRNA-130a-3p sponge
Source: Cell Death Discov. 2021 Dec 14;7:387. doi: 10.1038/s41420-021-00787-4 (PMC8671440; doi:10.1038/s41420-021-00787-4)
Supplement: Supplementary file 2 — Supplementary figure legends [file 41420_2021_787_MOESM2_ESM.docx]

**Supporting figures**

**Fig. 1** The expression levels of miRNA-130a-3p and miRNA-181a-5p in liver cancer tissues and normal tissues. **a** The expression of miRNA-181a-5p in liver cancer tissues and normal tissues was obtained through TCGA database. **b** The expression of miRNA-130a-3p in liver cancer tissues and normal tissues was obtained through TCGA database. ** and *** = *P* *<*0.01 and *<*0.001.

**Fig. 2** The expression of AR in HCC tissues and normal tissues was obtained through Oncomine database. *P* <0.001.
