# Supplementary figures and images for: LncRNA LINC00667 aggravates the progression of hepatocellular carcinoma by regulating androgen receptor expression as a miRNA-130a-3p sponge
Source: Cell Death Discov. 2021 Dec 14;7:387. doi: 10.1038/s41420-021-00787-4 (PMC8671440; doi:10.1038/s41420-021-00787-4)

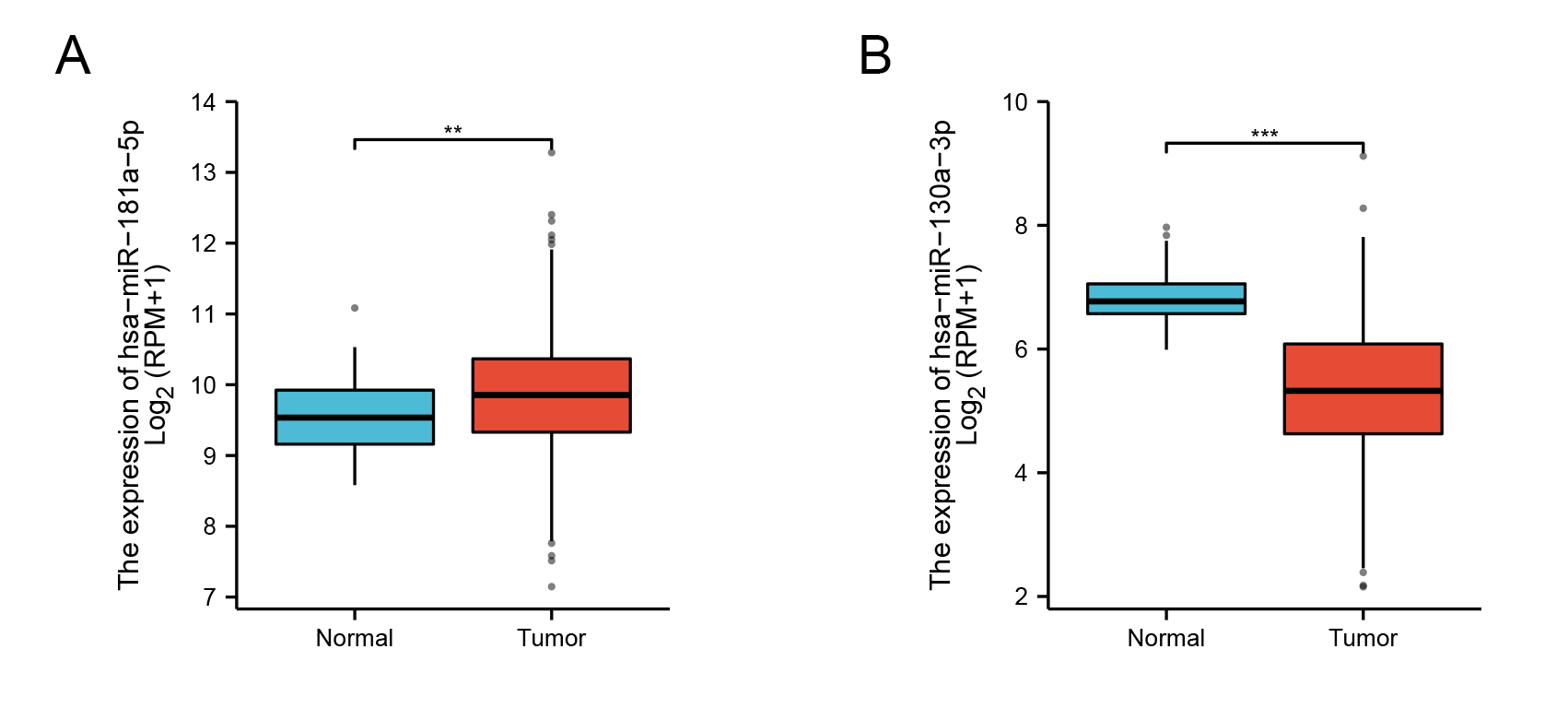

Supplement: Supplementary file 3 — Supplementary Figure 1 [file 41420_2021_787_MOESM3_ESM.tif]

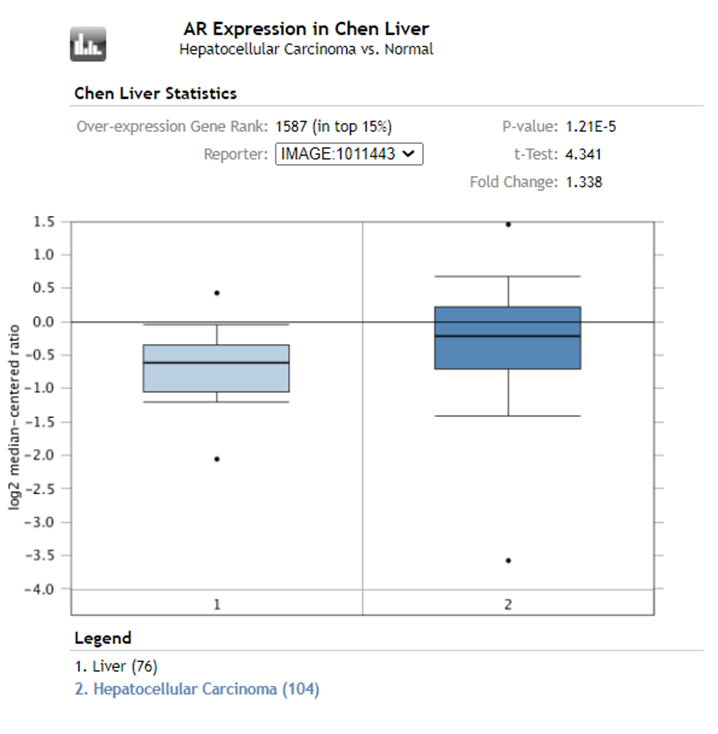

Supplement: Supplementary file 4 — Supplementary Figure 2 [file 41420_2021_787_MOESM4_ESM.tif]
