# Supplementary material for: LncRNA LINC00667 aggravates the progression of hepatocellular carcinoma by regulating androgen receptor expression as a miRNA-130a-3p sponge
Source: Cell Death Discov. 2021 Dec 14;7:387. doi: 10.1038/s41420-021-00787-4 (PMC8671440; doi:10.1038/s41420-021-00787-4)
Supplement: Supplementary file 5 — Supplementary Table 1 [file 41420_2021_787_MOESM5_ESM.docx]

**Supplementary Table 1. Candidate target genes of miRNA-130a-3p**

| SLMAP | RPS6KA5 | ACBD3 | SEL1L3 | UBE2W | CUL3 | ACSL1 | CLCN3 | SYBU |
| --- | --- | --- | --- | --- | --- | --- | --- | --- |
| MFSD6 | ACBD5 | NACC2 | PRKAA1 | SNX2 | PRUNE2 | PLAA | PSAP | AKAP1 |
| ZNF800 | RRAGD | AR | TRIM2 | CDS1 | ZBTB18 | HSPA8 | ITPRIPL2 | DNAJC16 |
| TEX2 | ARHGAP12 | ZBTB4 | MAPK1 | G3BP2 | UBE3B | SPTY2D1 | IMPDH1 | AKAP11 |
| INO80 | BHLHE41 | PPARG | SLC44A1 | UBXN2B | VPS13D | CLTC |  |  |
