# Supplementary material for: LncRNA LINC00667 aggravates the progression of hepatocellular carcinoma by regulating androgen receptor expression as a miRNA-130a-3p sponge
Source: Cell Death Discov. 2021 Dec 14;7:387. doi: 10.1038/s41420-021-00787-4 (PMC8671440; doi:10.1038/s41420-021-00787-4)
Supplement: Supplementary file 6 — Supplementary Table 2 [file 41420_2021_787_MOESM6_ESM.docx]

**Supplementary Table 2. Primer sequences and probe sequences**

| **Name** | **Sequence** |
| --- | --- |
| LINC00667-F | 5′-GTGGGTAGGAAACAGTCGGG-3′ |
| LINC00667-R | 5′-CTCAAAGGTGGCCAAAAGCC-3′ |
| GAPDH-F | 5′-GTCTCCTCTGACTTCAACAGCG-3′ |
| GAPDH-R | 5′-ACCACCCTGTTGCTGTAGCCAA-3′ |
| miR-130a-3p-F | 5'-TTCACATTGTGCTACTGTCTGC-3' |
| miR-130a-3p-R | 5'-GCTCTGACTTTATTGCACTACT-3' |
| U6-F | 5'-CTCGCTTCGGCAGCACA-3' |
| U6-R | 5'-AACGCTTCACGAATTTGCGT-3' |
| miR-130a-3p mimics | 5'-CAGUGCAAUGUUAAAAGGGCAU-3' |
| miR-130a-3p inhibitor | 5'-AUGCCCUUUUAACAUUGCACUG-3' |
| mimics-NC | 5'-UUCUCCGAACGUGUCACGUTT-3' |
| AR-F | 5'-TTCCCATTGTGGCTCCTATC-3' |
| AR-R | 5'-GTGGCTGGCACAGAGTAGTG-3' |
| miR-301a-3p-F | 5'-ACACTCCAGCTGGGCAGTGCAATAGTATTGTC-3' |
| miR-301a-3p-R | 5'-CTCAACTGGTGTCGTGGA-3' |
| miR-148a-3p-F | 5′-AGCAGTTCAGTGCACTACAG-3′ |
| miR-148a-3p-R | 5′-GCAGGGTCCGAGGTATTC-3′ |
| miR-181d-5p-F | 5′-GCAAACATTCATTGTTGTCGGT-3′ |
| miR-181d-5p-R | 5'-CCAGTGCAGGGTCCGAGGT-3' |
| miR-34a-5p-F | 5′-AGCCGCTGGCAGTGTCTTA-3′ |
| miR-34a-5p-R | 5'-CAGAGCAGGGTCCGAGGTA-3' |
| miR-181a-5p-F | 5'-GGGCAGCCTTAAGAGGA-3' |
| miR-181a-5p-R | 5'-CAGTGCGTGTCGTGGA-3' |
| miR-181b-5p-F | 5'-AACATTCATTGCTGTCGGTGGGT-3' |
| miR-181b-5p-R | 5'-GCGAGCACAGAATTAATACGAC-3' |
| miR-454-3p-F | 5'-GCGCGTAGTGCAATATTGCTTA-3' |
| miR-454-3p-R | 5'-AGTGCAGGGTCCGAGGTATT-3' |
| LINC00667 probe | 5'CY3-CAGGACGGGGCTCTCCCTCTCGATGT-3' |
|  |  |
