# Supplementary material for: LncRNA LINC00667 aggravates the progression of hepatocellular carcinoma by regulating androgen receptor expression as a miRNA-130a-3p sponge
Source: Cell Death Discov. 2021 Dec 14;7:387. doi: 10.1038/s41420-021-00787-4 (PMC8671440; doi:10.1038/s41420-021-00787-4)
Supplement: Supplementary file 7 — Supplementary Table 3 [file 41420_2021_787_MOESM7_ESM.docx]

**Supplementary Table 3. Antibodies used in this study**

| **Antibody** | **WB** | **IHC** | **Specificity** | **Company** |
| --- | --- | --- | --- | --- |
| AR (22089-1-AP) | 1:1000 | 1:100 | Mouse polyclonal | Proteintech |
| GAPDH (ab8245) | 1:5000 |  | Mouse monoclonal | Abcam |
| Slug (GTX128796) | 1:5000 | 1:200 | Rabbit polyclonal | GeneTex |
| Active-β-Catenin (05-665) | 1:1000 | 1:100 | Mouse monoclonal | MERCK |
| β-Catenin (ab32572) | 1:5000 | 1:500 | Mouse monoclonal | Abcam |
| E-cadherin (bs-1519R) | 1:500 | 1:200 | Rabbit polyclonal | Bioss |
| Vimentin (ab92547) | 1:2000 | 1:400 | Rabbit monoclonal | Abcam |
| Ki67 (NBP2-22112) |  | 1:400 | Mouse monoclonal | Novus |
